# Sex-Specific Cannabidiol- and Iloperidone-Induced Neuronal Activity Changes in an In Vitro MAM Model System of Schizophrenia

**DOI:** 10.3390/ijms22115511

**Published:** 2021-05-24

**Authors:** Rachel-Karson Thériault, Myles St-Denis, Tristen Hewitt, Jibran Y. Khokhar, Jasmin Lalonde, Melissa L. Perreault

**Affiliations:** 1 Department of Molecular and Cellular Biology, University of Guelph, Guelph, ON N1G 2W1, Canada; theriauk@uoguelph.ca (R.-K.T.); stdenis@uoguelph.ca (M.S.-D.); thewitt@uoguelph.ca (T.H.); jlalon07@uoguelph.ca (J.L.); 2 Collaborative Program in Neuroscience, University of Guelph, Guelph, ON N1G 2W1, Canada; jkhokhar@uoguelph.ca; 3 Department of Biomedical Sciences, University of Guelph, Guelph, ON N1G 2W1, Canada

**Keywords:** schizophrenia, cannabidiol, iloperidone, haloperidol, electrophysiology, primary cortical neurons

## Abstract

Cortical circuit dysfunction is thought to be an underlying mechanism of schizophrenia (SZ) pathophysiology with normalization of aberrant circuit activity proposed as a biomarker for antipsychotic efficacy. Cannabidiol (CBD) shows potential as an adjunctive antipsychotic therapy; however, potential sex effects in these drug interactions remain unknown. In the present study, we sought to elucidate sex effects of CBD coadministration with the atypical antipsychotic iloperidone (ILO) on the activity of primary cortical neuron cultures derived from the rat methylazoxymethanol acetate (MAM) model used for the study of SZ. Spontaneous network activity measurements were obtained using a multielectrode array at baseline and following administration of CBD or ILO alone, or combined. At baseline, MAM male neurons displayed increased bursting activity whereas MAM female neurons exhibited no difference in bursting activity compared to sex-matched controls. CBD administered alone showed a rapid but transient increase in neuronal activity in the MAM networks, an effect more pronounced in females. Furthermore, ILO had an additive effect on CBD-induced elevations in activity in the MAM male neurons. In the MAM female neurons, CBD or ILO administration resulted in time-dependent elevations in neuronal activity, but the short-term CBD-induced increases in activity were lost when CBD and ILO were combined. Our findings indicate that CBD induces rapid increases in cortical neuronal activity, with sex-specific drug interactions upon ILO coadministration. This suggests that sex should be a consideration when implementing adjunct therapy for treatment of SZ.

## 1. Introduction

Schizophrenia (SZ) is a chronic psychiatric disorder characterized by positive (e.g., hallucinations) and negative (e.g., anhedonia) symptoms, as well as debilitating cognitive dysfunction [1,2]. Unfortunately, currently available antipsychotics do not adequately treat these cognitive deficits [3]. Our understanding of the disorder is further complicated by the presence of sex differences in SZ [4]. For instance, men exhibit an earlier onset and poorer course of the illness [4], while women show significantly greater improvement in overall symptom severity in response to antipsychotic treatment [5].

Cortical neuronal activity has been linked to symptoms of SZ [6,7,8]. As such, an emerging mechanism underlying the pathophysiology of SZ is impaired circuit activity [9]. Specifically, clinical and preclinical studies have consistently reported aberrant cortical synchrony, functional connectivity, or laterality in SZ, and these abnormalities have been associated with cognitive deficits [10,11] and positive symptoms [8]. For instance, in a task of executive functioning and sensory processing, individuals with SZ displayed impaired cortical activity within the gamma (30–80 Hz) and theta (4–8 Hz) frequency bands, compared to healthy controls [12,13,14,15,16,17]. Thus, normalization of impaired circuit function has been proposed as a biomarker of therapeutic efficacy of antipsychotics [18,19]. This idea is supported by studies demonstrating that antipsychotic treatments normalize deficits in cortical activity in individuals with SZ [18] and in animal models of SZ [17,19,20,21]. Recently, our laboratory demonstrated that the degree of antipsychotic-induced network activity normalization in the methylazoxymethanol acetate (MAM) rat model system of SZ was greater with the administration of the atypical antipsychotic asenapine maleate, compared to the typical antipsychotic haloperidol (HAL) [22]. In line with this, the degree of normalization may positively correlate with improved cognitive functioning [18,19], an effect that is more prominent with atypical antipsychotics than typical antipsychotics [23]. This difference in efficacy between these two drug classes is postulated to be due to the increased affinity of atypical antipsychotics for serotonin over dopamine receptors [23]. 

Although current antipsychotics successfully treat acute positive symptoms, long-term improvement of other symptoms, particularly negative symptoms, is low [24]. Furthermore, some atypical antipsychotics have been found to impair cognitive function in a dose-dependent manner [25,26]. As such, poor treatment responses, in addition to poor insight due to illness, are the biggest contributors to the high rate of treatment non-adherence in SZ [24,27,28,29]. Thus, it is imperative that safe and effective treatment strategies are developed that show improved efficacy and compliance. 

One promising approach has been the use of the non-psychoactive cannabinoid cannabidiol (CBD) as an adjunctive therapy for the treatment of SZ [30,31,32,33,34]. For instance, a recent study demonstrated that when individuals with SZ were given adjunctive CBD to their current antipsychotic treatment, there was an increased tolerability profile and efficacy of positive and cognitive symptom management [34]. Moreover, it has been suggested that CBD adjunctive therapy may be particularly suitable in patients that exhibit antipsychotic resistance after a case study reported the full remission of an individual with severe, antipsychotic resistant SZ following adjunctive CBD [35]. As such, CBD may complement antipsychotic treatments due to its different mechanism of action [35]. Conversely, it has also been reported that 6 weeks of added CBD treatment had no significant effect on antipsychotic-treated individuals with chronic SZ [36]. Thus, additional investigations are necessary to elucidate the effectiveness of adjunctive CBD with antipsychotics in SZ. Importantly, the impact of adjunctive CBD on antipsychotic-induced normalization of network activity in SZ is unknown. 

In the present study, we sought to evaluate the effects of CBD adjunctive to the atypical antipsychotic iloperidone (ILO) on the activity of rat primary cortical neurons derived from the MAM model system of SZ and to determine whether sex-dependent effects could be detected. Comparisons to the traditional antipsychotic HAL were also performed. The findings showed that there exists sex- and model-specific drug-induced changes in neuronal systems activity, indicating that sex should be an important consideration when employing adjunct therapy, as drug interaction effects may be different.

## 2. Results

### 2.1. MAM Rats Exhibit Sex-Specific Differences in Baseline Neuronal Activity

**Figure 1:**
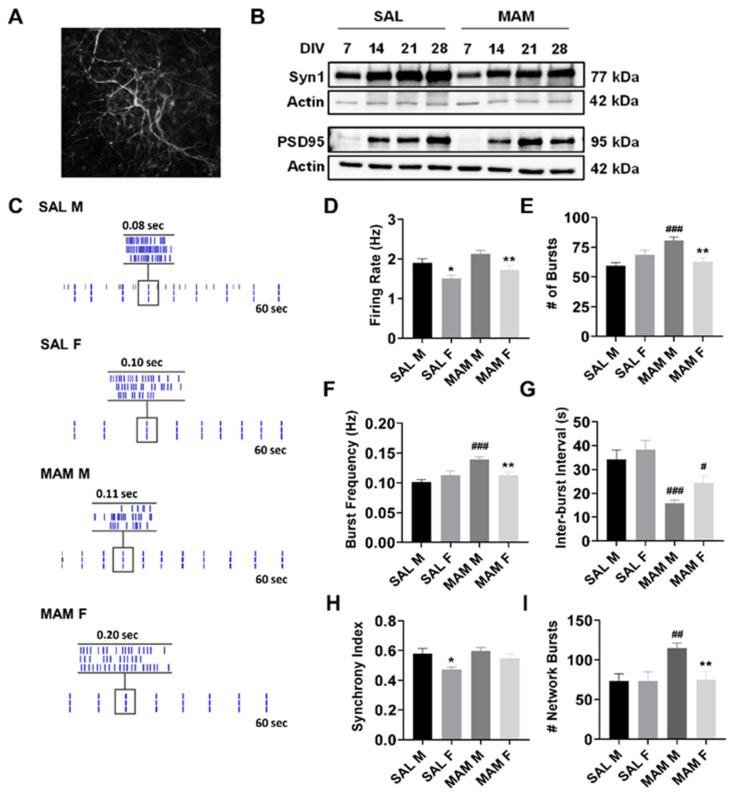
Effect of MAM on neuronal cortical systems activity. (**A**) Representative immunostaining of MAP2 showing a DIV 21 primary cortical neuron. (**B**) Western blot showing levels of Syn1, PSD-95 and actin at DIV 7, 14, 21, and 28 in primary cortical neurons derived from male and female SAL treated (control) and MAM rats. (**C**) Representative raster plots showing sex differences in baseline neuronal activity. (**D**) Quantification of group differences in neuronal firing rate showed that female SAL and MAM rat neurons exhibited reduced firing rates at baseline compared to male neurons of the same model. (**E**,**F**) Male MAM-derived neurons exhibited increased number of bursts compared to male SAL neurons, whereas female MAM neurons exhibited no difference in bursting activity compared to female SAL neurons. (**G**) Inter-burst interval showing a shorter interval in MAM-derived neurons. (**H**) Female SAL neurons showed a decreased synchrony index at baseline. (**I**) Male MAM-derived neurons exhibited elevated network bursting compared to all other groups. Data are expressed as means ± SEM. N = 3 biological replicates. * *p* < 0.05, ** *p* < 0.01, compared to males of the same model, ^#^ *p* < 0.5, ^##^ *p* < 0.01, ^###^ *p* < 0.001 compared to sex-matched controls, ANOVA followed by Bonferroni or Games–Howell post-hoc determined by Levene’s test of variance.

In the present study, we sought to elucidate the impact of CBD administration alone, or with ILO, on neuronal systems activity in vitro in cortical neurons derived from the MAM model system of SZ, and to evaluate sex differences. Using cortical neuron-glial co-cultures, we showed that neurons developed normally over the course of 4 weeks (Figure 1A,B), with a transient increase in the expression of both synapsin 1 (Syn1) and postsynaptic density 95 (PSD-95) proteins. Multielectrode array (MEA) recordings were performed on day 21 with raster plots showing baseline activity for each group (Figure 1C). Analysis of the firing rate (Figure 1D) showed significant sex and model effects (Sex: F(1, 1156) = 17.1, *p* < 0.001; Model: F(1, 1156) = 7.0, *p* < 0.008). Female SAL neurons innately displayed lower cortical neuronal firing compared to control males (*p* = 0.036), an effect also observed between the MAM female versus MAM male neurons (*p* = 0.009). Conversely, no difference in the neuronal firing rate was evident between groups of the same sex. There was no innate difference in bursting activity between male and female SAL neurons (Figure 1E,F). However, neurons from male MAM rats exhibited elevated bursting activity compared to their SAL control counterparts (*p* < 0.001 for both number of bursts and bursting frequency). The female MAM neurons did not show this elevation in bursting and were not different from the female SAL neurons. There were no sex differences observed in the inter-burst interval with the SAL neurons (Figure 1G). However, both male (*p* < 0.001) and female (*p* = 0.029) MAM-derived neurons showed a reduced interval compared to their same-sex counterparts (Number of Bursts. Model: F(1, 1156) = 10.6, *p* = 0.001; Sex x Model: F(1, 1156) = 12.1, *p* = 0.001, Burst Frequency. Model: F(1, 1104) = 13.5, *p* < 0.001; Sex x Model: F(1, 1104) = 11.6, *p* = 0.001, Inter-burst Interval. Model: F(1, 1072) = 26.4, *p* < 0.001). We next evaluated network activity and showed a lower synchrony index in the female SAL neurons compared to the other groups, and with no other group differences observed (Figure 1H, Sex: F(1, 86) = 8.0, *p* = 0.006). Male MAM-derived neurons showed significantly elevated network bursts compared to all other groups, with no other group differences present (Figure 1I, Sex: F(1, 92) = 4.5, *p* = 0.037; Model: F(1, 92) = 5.3, *p* = 0.023; Sex x Model: F(1, 92) = 4.50, *p* = 0.037).

### 2.2. Drug-Induced Effects on Neuronal Activity in Male Control Rat Cortical Neurons

**Figure 2:**
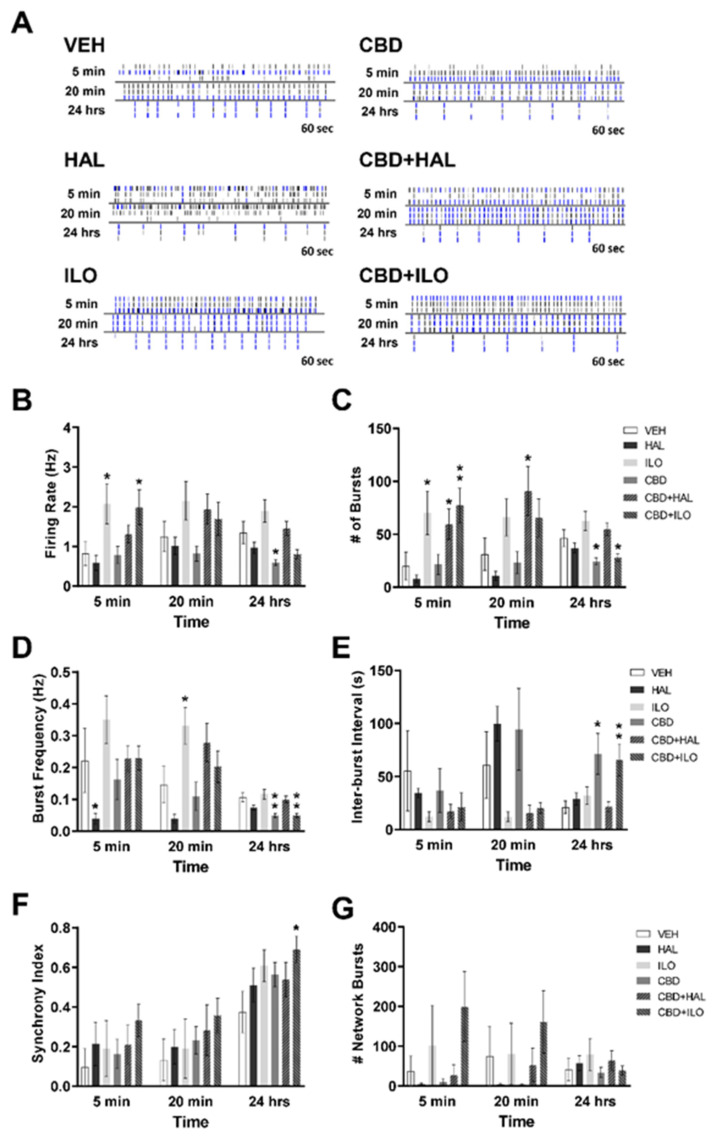
Temporal effect of cannabidiol (CBD) and/or antipsychotic exposure on neuronal activity in cultures derived from male SAL rats. (**A**) Representative raster plots showing neuronal activity following administration of each drug at 5 min, 20 min, and 24 h. (**B**) CBD or CBD+iloperidone (ILO) elevated spiking activity at 5 min, with a reduction in spiking by CBD at 24 h. (**C**) CBD alone or combined with haloperidol (HAL) or ILO increased the number of neuronal bursts at 5 min, an effect maintained in the CBD+HAL group at 20 min. At 24 h, CBD or CBD+ILO groups showed reduced number of bursts. (**D**) HAL administration reduced burst frequency at 5 min, whereas ILO increased bursting frequency at 20 min. At 24 h, CBD or CBD+ILO administration suppressed bursting frequency. (**E**) CBD alone, or with ILO, increased the inter-burst interval at 24 h. (**F**,**G**) Only CBD+ILO increased the synchrony index in male SAL neurons with no drug effects on the number of network bursts. Data are expressed as means ± SEM. N = 3 biological replicates. * *p* < 0.05, ** *p* < 0.01 compared to VEH-exposed neurons, planned comparison Student’s *t*-test.

We next sought to elucidate temporal drug-induced changes in neuronal systems activity for each of the groups. Raster plots for the male SAL neurons are depicted in Figure 2A. ANOVA revealed a significant drug effect for the firing rate at 5 min (F(5, 286) = 3.8, *p* = 0.002). Administration of ILO or CBD+ILO increased the mean firing rate of cortical neurons within 5 min with effects lost by 20 min, such that there was no difference when compared to VEH administration (Figure 2B). A CBD-induced reduction in the firing rate of neurons was observed 24 h post-administration (*p* = 0.011). Similarly, ILO or CBD+ILO, or CBD+HAL, increased the number of bursts at 5 min (F(5, 286) = 4.8, *p* < 0.001), with CBD or CBD+ILO inducing a decrease in burst number (F(5, 287) = 7.0, *p* < 0.001) and burst frequency (F(5, 246) = 8.5, *p* < 0.001) at 24 h (Figure 2C,D). CBD or CBD+ILO also increased the inter-burst interval at 24 h post-administration compared to VEH (*p* = 0.016, *p* = 0.008, respectively, Figure 2E). When network activity was evaluated, only CBD+ILO resulted in an elevated synchrony index in the SAL male neurons (*p* = 0.044), with no drug effects on the number of network bursts at any time point (Figure 2F,G). 

### 2.3. Drug-Induced Effects on Neuronal Activity in Female Control Rat Cortical Neurons

**Figure 3:**
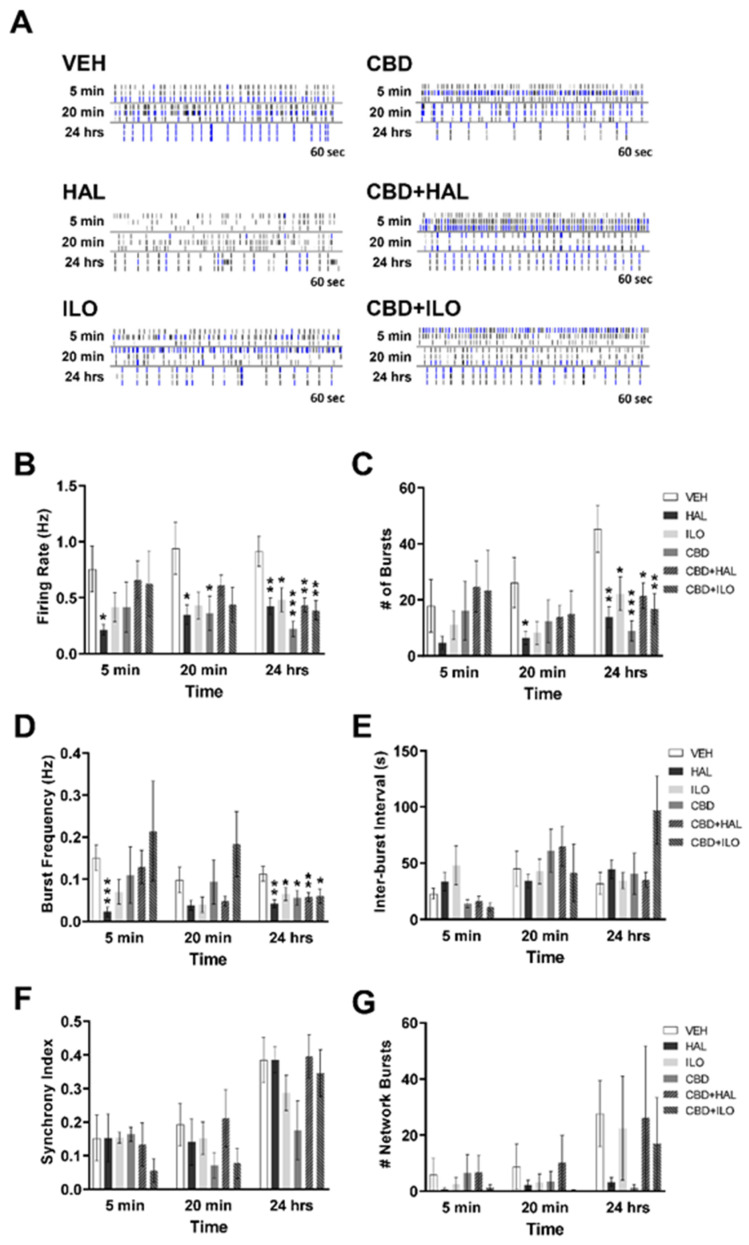
Temporal effect of cannabidiol (CBD) and/or antipsychotic exposure on neuronal activity in cultures derived from female SAL rats. (**A**) Representative raster plots following administration of each drug at 5 min, 20 min, and 24 h. (**B**–**D**) All drugs induced a transient decrease in firing rate, number of bursts, and burst frequency with all drugs inducing significantly reduced activity at 24 h. However, haloperidol (HAL) showed rapid onset effects, decreasing firing and bursting activity at 5 min. (**E**–**G**) There were no drug effects on inter-burst interval, synchrony index, or the number of network bursts at any time point. Data are expressed as means ± SEM. N = 3 biological replicates. * *p* < 0.05, ** *p* < 0.01, *** *p* < 0.001 compared to VEH-exposed neurons, planned comparison Student’s *t*-test.

Drug effects on neuronal activity in the female SAL neurons were next evaluated with group raster plots, shown in Figure 3A. All drugs induced a transient decrease in the firing rate, number of bursts, and burst frequency, with all drugs inducing significantly reduced activity by 24 h (Figure 3B–D, Drug effects. Firing rate: (F(5, 373) = 2.2, *p* < 0.053; Number of bursts: (F(5, 373) = 5.5, *p* < 0.001); Burst frequency: (F(5, 354) = 4.8, *p* < 0.001). HAL appeared to induce these effects more rapidly, with reduced firing and bursting activity apparent within 5 min. There were no group differences in the inter-burst interval, synchrony index, or the number of network bursts (Figure 3E–G). 

### 2.4. Drug-Induced Effects on Neuronal Activity in Male MAM Rat Cortical Neurons

**Figure 4:**
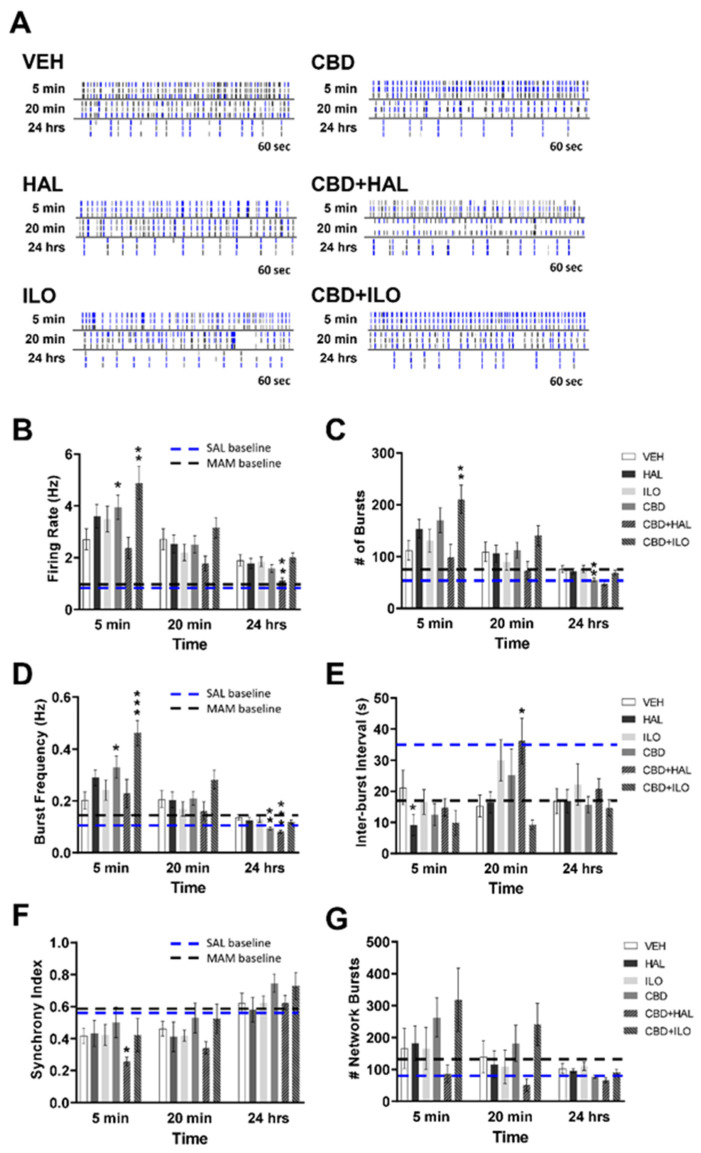
Temporal effect of cannabidiol (CBD) and/or antipsychotic exposure on neuronal activity in cultures derived from male MAM rats. (**A**) Representative raster plots following administration of each drug at 5 min, 20 min, and 24 h. (**B**) Compared to VEH-exposed neurons, at 5 min CBD increased the spiking rate, an effect exacerbated in the presence of iloperidone (ILO). At 24 h, CBD+haloperidol (HAL) administration lowered the spiking rate. (**C**) CBD+ILO increased the number of bursts at 5 min, with CBD alone suppressing burst number at 24 h. (**D**) CBD or CBD+ILO increased the bursting frequency at 5 min, with CBD or CBD+HAL lowering it at 24 h. (**E**) HAL alone resulted in a lower inter-burst interval at 5 min, whereas CBD+HAL elevated it at 20 min. (**F**) CBD+HAL suppressed the synchrony index at 5 min. (**G**) There were no drug effects on the number of network bursts. SAL- and MAM-derived baseline data is depicted by the blue and black dashed lines, respectively. Data are expressed as means ± SEM. N = 3 biological replicates. * *p* < 0.05, ** *p* < 0.01, *** *p* < 0.001 compared to VEH-exposed neurons, planned comparison Student’s *t*-test.

The impact of CBD and/or antipsychotic administration on neuronal systems activity was next determined, with raster plots for the male MAM neurons shown in Figure 4A. There were significant drug effects evident at 5 min for neuronal firing rate (F(5, 321) = 3.5, *p* = 0.004), number of bursts (F(5, 321) = 3.4, *p* = 0.005), and burst frequency (F(5, 268) = 5.2, *p* < 0.001). At 5 min, CBD was the only drug, when administered alone, that affected neuronal activity (Figure 4B–D), with the exception of HAL, which reduced the inter-burst interval compared to VEH treatment (*p* = 0.037, Figure 4E). Specifically, CBD administration resulted in a higher firing rate (Figure 4B) and burst frequency (Figure 4D) of male MAM cortical neurons, with these effects exacerbated with the addition of ILO. There were no effects on the number of bursts or inter-burst interval by CBD alone; however, CBD+ILO resulted in a significant elevation in the number of bursts (Figure 4C,E). At 24 h, CBD suppressed the number of bursts and burst frequency, similar to that observed in the male and female SAL neurons. However, these effects were not seen in the CBD+ILO group at 24 h. For inter-burst interval, no effects were observed with any drug at any time points except for HAL alone, which induced a decrease at 5 min (*p* = 0.041), and CBD+HAL, which induced an increase in the inter-burst interval at 20 min (*p* = 0.035). It is also noteworthy that, unlike that observed in the SAL control neurons, when compared to baseline (Figure 1), only the addition of CBD or CBD+ILO induced an overall increase in neuronal activity in all three measures—firing rate, number of bursts, and burst frequency—at 5 min (*p* < 0.001 for each measure, in both groups), effects that diminished by 24 h. Network activity analysis showed no drug-induced differences (Figure 4F,G). Together, these findings demonstrate the additive effects of CBD+ILO administration in the male MAM neurons. 

### 2.5. Drug-Induced Effects on Neuronal Activity in Female MAM Rat Cortical Neurons

**Figure 5:**
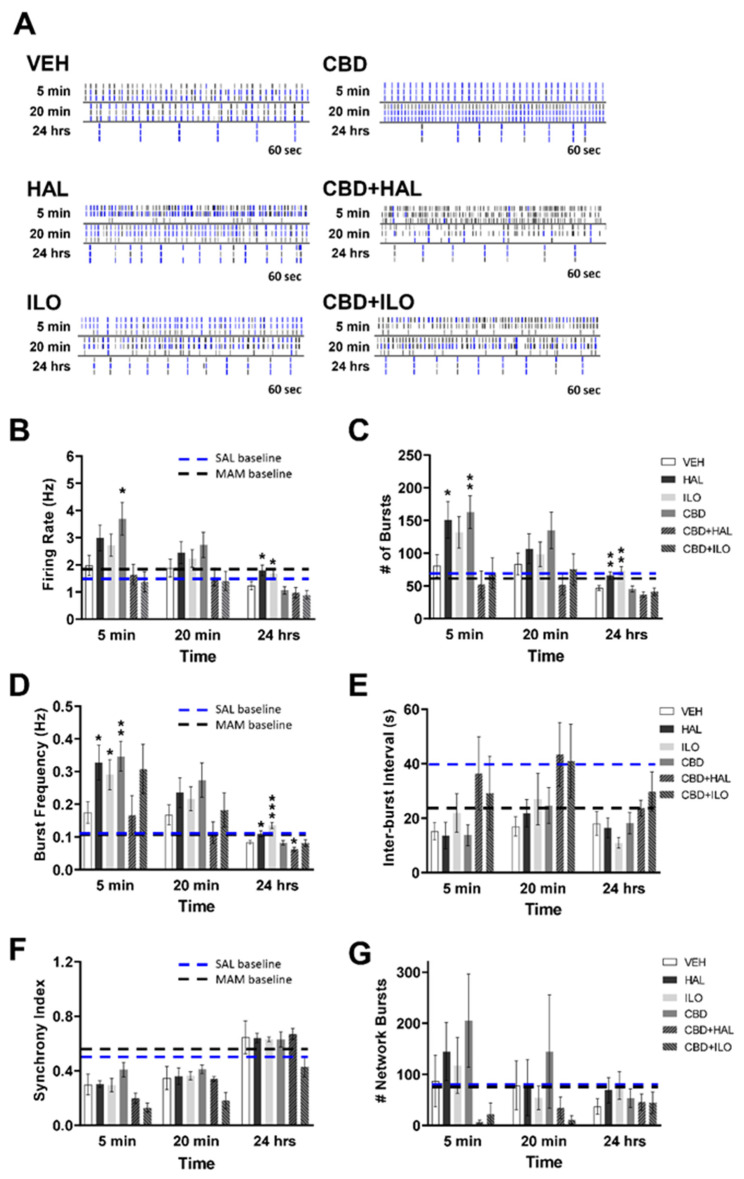
Temporal effect of cannabidiol (CBD) and/or antipsychotic exposure on neuronal activity in cultures derived from female MAM rats. (**A**) Representative raster plots following administration of each drug at 5 min, 20 min, and 24 h. (**B**) CBD exposure resulted in a higher firing rate at 5 min compared to VEH. Haloperidol (HAL) or iloperidone (ILO) increased the firing rate at 24 h. (**C**,**D**) HAL or CBD alone resulted in an elevated number of bursts and bursting frequency at 5 min. ILO administration also elevated the bursting frequency at this time point. At 24 h, HAL or ILO elevated both the number of bursts and the bursting frequency. (**E**–**G**) There were no drug effects observed at any time point on the inter-burst interval, synchrony index, or number of network bursts. SAL- and MAM-derived baseline data is depicted by the blue and black dashed lines, respectively. Data are expressed as means ± SEM. N = 3 biological replicates. * *p* < 0.05, ** *p* < 0.01, *** *p* < 0.001 compared to VEH-exposed neurons, planned comparison Student’s *t*-test.

Finally, we examined drug effects in the female MAM neurons with raster plots showing neuronal activity, depicted in Figure 5A. In these neurons, we observed significant short-term effects of CBD on neuronal activity, with CBD-induced elevations in firing rate (*p* = 0.017), burst number (*p* = 0.006), and burst frequency (*p* = 0.004) at 5 min compared to VEH-treated MAM neurons (Figure 5B–D, Drug effects. Firing rate: F(5, 249) = 4.1, *p* = 0.001; Number of bursts: F(5, 249) = 4.7, *p* < 0.001; Burst frequency: F(5, 157) = 2.4, *p* < 0.04). However, when ILO was co-administered, these CBD-induced effects were abolished. HAL or ILO alone had similar effects on neuronal activity, increasing neuronal firing and/or bursting activity at 5 min, effects also seen at 24 h (Drug effects. Firing rate: F(5, 252) = 5.0, *p* < 0.001, Number of bursts: F(5, 252) = 9.8, *p* < 0.001, Burst frequency: F(5, 226) = 10.3, *p* < 0.001). There were no drug effects observed on the inter-burst interval (Figure 5E) or on network activity (Figure 5F,G). When compared to baseline (Figure 1), the addition of CBD or ILO increased the firing rate (CBD: *p* = 0.04; ILO, *p* = 0.02), number of bursts (CBD: *p* = 0.001; ILO, *p* = 0.03), and burst frequency (CBD: *p* < 0.001; ILO, *p* = 0.002) at 5 min, which returned to baseline levels by 24 h. These findings demonstrate that CBD or antipsychotics alone had the most robust effects in the female MAM neurons; however, these effects were attenuated when the drugs were combined.

## 3. Discussion

The present study aimed to delineate the temporal and sex-dependent effects of acute CBD, alone or in combination with HAL or ILO, on neuronal activity of primary cortical neurons derived from the MAM neurodevelopmental animal model of SZ. We demonstrated that at baseline, exposure to MAM in the dams significantly increased bursting activity in primary neurons derived from the male offspring, while female offspring-derived cultures exhibited no change, compared to their sex-matched SAL control groups. When administered alone, CBD induced a rapid and transient increase in neuronal activity in the MAM-derived cultures. Further, this transient increase in neuronal activity was additive when CBD was combined with ILO in MAM male neurons only. Conversely, in MAM females, co-administration of CBD with ILO abolished the effects observed when CBD was given alone. Collectively, these results suggest that CBD may elicit rapid effects through increased neuronal activity, although adjunctive CBD therapy may have differential effects in males and females. Thus, sex should be a consideration when employing combination therapy. 

Baseline recordings of cortical neurons indicated sex differences in the activity of MAM-derived cultures, whereby males showed increased bursting while both males and females exhibited a decreased inter-burst interval, relative to sex-matched control cultures. The majority of studies have reported reduced spontaneous and evoked network activity, mean firing frequencies and average burst duration in cortical [37,38,39] and hippocampal (HIP) human-induced pluripotent stem cells (hiPSC) derived from SZ patients [40]. However, evidence of increased bursting and spontaneous network activity has also been found in SZ mouse model-derived HIP cultures [41] and SZ patient-derived cortical hiPSCs [42]. Notably, these studies did not examine whether the results were influenced by sex; therefore, we propose that the sex-dependent effects in our study may provide insights into the conflicting reports thus far. 

It is well documented that there are prominent sex differences in SZ at both the clinical and physiological levels [4,43,44]. The incidence of SZ in men to women is 1.4:1 and the peak age of onset for SZ in men is in their early twenties. In women, although the incidence is less than that of men, the peak age of onset is later, being highest in their twenties and with an additional peak in onset in midlife, where women exhibit a higher incidence than males [4,45]. Men are also known to exhibit a more severe phenotype of the disease, with more severe negative and cognitive symptoms compared to women, whereas women exhibit more severe mood-related symptoms [4]. The long-term prognosis for SZ also differs between men and women, with women displaying superior long-term outcomes and responses to antipsychotic treatment [4,46]. Furthermore, adverse side effects of antipsychotic medications are also more prevalent in women [47]. Evidence from individuals with SZ also indicates that there are sex-dependent genetic risk factors [44], and sex differences in the serum concentrations of multiple molecules associated with inflammatory, hormonal, and growth factor signalling pathways that have been correlated to symptom severity [43]. Sex differences at the cellular and molecular level have also been reported in animal models of SZ. In the prenatal immune challenge model of SZ in mice, for example, distinct sex differences were found in microglial distribution, process arborization, oxidative stress, synaptic function, and expression of inflammatory genes that may be relevant to the increased incidence of severe SZ in males [48]. In the MAM model of SZ in mice, sex differences have been reported not only in neuronal activity, but also in the expression of regulatory proteins, primarily those involved in glutamatergic signaling in the prefrontal cortex [49]. While glutamatergic transmission drives excitation in cortical circuits, inhibition from gamma aminobutyric acid (GABA)ergic interneurons plays a key role in regulating excitability of pyramidal neurons in order to generate synchronized firing patterns required for efficient communication between neuronal ensembles [11]. Post-mortem analysis of the cingulate cortex of individuals with SZ revealed decreased and increased expression levels of GABAergic genes in men and women, respectively, compared to sex-matched controls [50]. Further, cortical GABAergic interneurons that express parvalbumin, critical regulators of activity balance in SZ [37], are reduced in number [51]. To our knowledge, sex differences in parvalbumin expression have not yet been evaluated in humans, although some findings have shown sex-specific differences in the MAM rodent model of SZ. For example, a decrease in parvalbumin-positive interneurons, in line with those observed in human SZ patients, in both MAM-treated male and female rats has been shown [10,52]. Conversely, Chalkiadaki et al. [49] found that MAM exposure decreased the expression of parvalbumin in male but not in female mice, and that only male mice exhibited prefrontal cortical deficits in activity, a discrepancy that may be explained by the different species employed. 

In this study, we observed sex- and model-specific effects in response to CBD administration alone. Both male and female MAM-derived cultures displayed rapid increases in neuronal activity, as evidenced by an elevation in the mean firing rate, number of bursts, and burst frequency in response to CBD that were absent in the SAL culture neurons. Interestingly, these effects were also present with ILO alone in the female MAM cultures. These findings may not only indicate potentially increased sensitivity of the MAM cultures to CBD, but a sex-specific sensitivity to ILO. At 24 h post-administration, however, decreased activity was evident in both male MAM- and SAL-derived cultures. In the female neurons, only the SAL-derived cultures showed suppressed activity, which occurred as early as 20 min post-drug exposure. These results indicate that CBD may have unique effects in healthy individuals versus those with an underlying disorder such as SZ and, further, that this may occur in a sex-specific manner. Despite evidence of sex differences in the effects of cannabinoids such as THC, evidence of sex differences in the biological responses to CBD are lacking [53]. Additionally, evidence of CBD effects on network activity is also sparse. However, electroencephalographic (EEG) and functional magnetic resonance imaging (fMRI) studies have reported altered functional connectivity patterns and network integration following a month of CBD treatment in healthy participants [54,55]. Although the functional relevance of these findings is unknown, they support CBD-induced alterations in synchronized neural activity [54]. To our knowledge, our findings are the first to demonstrate transient increases in neuronal activity following CBD administration in cortical neurons derived from the MAM model of SZ, and we postulate that these alterations may be linked to the rapid physiological effects known to accompany CBD exposure [56,57]. Chronic CBD monotherapy has been demonstrated to exert antipsychotic properties in clinical studies [33,58], as well as ameliorate social and cognitive deficits in rodent models of SZ [59,60,61,62]. 

Interestingly, when administered alone, HAL and ILO showed minimal to no significant effect on neuronal activity in MAM male neurons but increased burst frequency, number of bursts, and firing rate in MAM female neurons. This finding may suggest a greater biological impact of antipsychotics in women than in men, and is in line with clinical findings showing that women respond better to antipsychotic treatment than men [4]. Further, this antipsychotic-induced increase in neuronal activity supports a previous MEA study in primary cortical neurons of wild-type (WT) mice, which reported that acute therapeutic doses of typical and atypical antipsychotics increased network-wide temporal synchronization [63]. Other MEA studies have found increased neuronal activity and synchronization with atypical antipsychotics only, and alterations in the opposite direction occurring with HAL exposure [64,65]. However, this discrepancy is likely a result of these experiments having been conducted in rodent primary HIP cultures and/or the model system used [64,65]. Interestingly, the enhanced activity in MAM femalecultures was maintained 24 h post-administration of antipsychotics, with trends evident at 20 min. This is in line with past evidence of antipsychotic-induced increases in neuronal activity at 10 min post-administration [63], and follows the pharmacological timelines observed in clinical studies. Specifically, within 24 h of receiving HAL or atypical antipsychotic medication, individuals with SZ displayed significantly increased dopamine receptor blockade [66,67], as well as improved positive symptoms [68]. Therefore, it is possible that the network alterations we observed at 24 h post-administration are therapeutically relevant. 

Evidence suggests that positive long-term effects of antipsychotic drugs may be attributed to changes in synaptic plasticity [69,70]. For instance, antipsychotics are thought to induce changes in synaptic function, and subsequent network activity, through modulating the expression of synaptic proteins, dendritic branch structure, and spine density [64,65,70,71]. Evidence of these effects are most understood in the context of chronic treatment; however, there is evidence of acute exposure to antipsychotics modifying dendritic morphology, thereby influencing synaptic function. Acute administration of HAL and atypical antipsychotics was shown to increase dendritic complexity in primary striatal neurons after 24 h [72], and alterations in dendritic complexity of pyramidal neurons has been associated with modified neuronal activity [73,74,75]. Acute treatment with the atypical antipsychotic olanzapine was also shown to reverse spine density deficits in an animal model of SZ and required chronic treatment to maintain this effect [76]. Furthermore, studies of immediate-early genes (IEGs) suggest that the activation of IEGs with acute antipsychotic treatment plays a key role in mediating their acute therapeutic effects and may set in motion the long-term changes associated with chronic treatment [77]. Although in the present study, increased activity with HAL and ILO administration was observed in MAM-derived female cultures only, this discrepancy could be explained due to many of these studies modeling repeated exposure to antipsychotic drugs over multiple days. It is noteworthy, however, that this observation in MAM female cultures could indicate that females are also more sensitive to the sustained effects of antipsychotic drugs.

As previously mentioned, adjunctive CBD therapy is a promising new approach for the treatment of SZ. Recent clinical studies have found significantly improved medication tolerability, positive symptoms, and cognitive dysfunction, with a trend of ameliorated negative symptoms, in SZ patients given adjunctive CBD [34,35]. However, support for a lack of significant effect of CBD on antipsychotic-treated individuals with chronic SZ has also been reported [36]. Our results suggest that the discrepancy in findings may be, at least in part, tied to sex differences. Specifically, in male MAM neurons, the combination of CBD with ILO appeared to have an additive effect, with a rapid and transient increase in neuronal activity having been observed. Although CBD with HAL was observed to induce a sustained decrease in bursting activity approaching baseline activity in SAL males at 24 h, this could simply be attributed to the effects of CBD, as this was also observed with CBD administration alone and not HAL. Conversely, while exposure to CBD or antipsychotics alone caused a transient increase in activity in MAM females, combination administration abolished the effects of either drug at all time points. Taken together, these results may indicate that CBD adjunctive therapy is more beneficial in males than females, although this hypothesis assumes that the increased neuronal activity is indeed therapeutic. As such, future studies are warranted to link these drug-induced changes in network activity with behavioural output. 

It is worth noting that cortical neuron-glial co-cultures were utilized in the present study to better emulate in vivo conditions. It is well known that SZ is associated with neuroinflammation and the elevation of pro-inflammatory cytokines is associated with more severe presentation of symptoms [78,79,80,81]. HAL, atypical antipsychotics, and CBD have all been demonstrated to inhibit the release of pro-inflammatory cytokines in vitro [82,83,84,85,86,87]. The anti-inflammatory effects of these drugs, and CBD in particular, are thought to account in part for their therapeutic effects [60,82,88]. Activation of glial cells and subsequent pro-inflammatory cytokine cascades are known to influence the activity and function of neural networks through modulation of synaptic plasticity [89,90,91]. Furthermore, both microglia and astrocytes are now known to play a key role in regulating synaptic transmission [92,93,94,95,96]. Microglia have been shown to detect synaptic activity and modulate synaptic remodelling through multiple mechanisms [94,95,96]. Notably, microglial secretion of the cytokine TNF-α is known to play a key role in synaptic scaling, a key mechanism for maintaining homeostatic activity through modulation of glutamatergic signalling [95,96,97]. Although it is unclear whether CBD produces antipsychotic effects mediated through microglia in the absence of pathological microglial activation, the effects of CBD monotherapy observed in the present study may be partly attributed to its effects on the functioning of astrocytes. Although the mechanisms by which CBD modulates the activity of astrocytes are not well understood at this time, CBD has been shown to increase production of the endogenous cannabinoid anandamide [33], which is known to activate CB1 receptors to increase the release of glutamate from astrocytes, thus increasing glutamatergic transmission [93,98,99]. Indeed, the increased expression of CB1 receptors has been found in patients with SZ and in the prefrontal cortex of MAM model rats [100]. 

Although this study provides important insights into sex differences in CBD and antipsychotic effects, it is important to highlight limitations associated with our experimental approach. First, this study was conducted in vitro, which does not mimic the environment and intricate neuronal networks that are present in in vivo model systems. For example, structural and functional laterality are hallmarks of SZ [8,101] that may also exhibit sex differences [101] and which cannot be captured in in vitro systems. Second, the behavioural output associated with the changes in neuronal activity cannot be assessed and therefore, the functional relevance can only be postulated. Third, only short-term drug effects were examined and chronic treatment of SZ is required in the clinical setting [102]. Consequently, whether the alterations we observed with acute CBD and antipsychotics are sustained with long-term treatment is not known and should be the focus of future studies. In summary, it is clear that more studies are required to fully elucidate the impact of adjunctive CBD therapy on network activity in SZ and the associated sex differences and behavioural effects. In this study, our findings suggest that CBD or ILO administration alone may be more relevant in women with SZ in the context of neuronal activity, while men with SZ may benefit more from CBD adjunctive therapy with ILO. This highlights that sex should be included as a critical factor when determining treatments and that sex differences should be assessed in future studies. 

## 4. Materials and Methods 

### 4.1. Animals

Six pregnant Sprague-Dawley rats (Charles River, QC, Canada) were injected with MAM (22 mg/kg, intraperitoneally (i.p.)) or saline (SAL) at a volume of 1 mL/kg on gestational day 17, as previously described [22]. Rats were provided food and water ad libitum and maintained on a 12 h reverse light-dark cycle (0800 h lights off; 2000 h lights on). All procedures were in accordance with the guidelines defined by the Guide to the Care and Use of Experimental Animals (Canadian Council on Animal Care, 1993) and the Animal Care Ethics Committee of the University of Guelph (protocol number: AUP#3734, approved 18 January 2018).

### 4.2. Cell Culture

Cortical neuronal glial co-cultures were prepared from postnatal day 0–1 Sprague Dawley rat pups. Pups were euthanized by decapitation and their brains were dissected out of the skull in ice-cold dissecting media (1X HBSS, 1% 1M HEPES, 1% penicillin/streptomycin; all Gibco, Fisher Scientific, Mississauga, ON, Canada) in a glass petri dish. The meninges were removed and cerebral cortices were isolated and processed individually. Following three washes in dissecting media, the cortices were digested using 2.5% Trypsin (Gibco) at 37 °C for 20 min, inverting halfway through. The cortices were then washed twice in dissecting media, triturated, and passed through a 100 µm pore falcon cell strainer (Fisher Scientific, Ottawa, ON, Canada). Following centrifugation of the samples (200× *g* at room temperature), the supernatant was aspirated and the cells were resuspended in plating media (Neurobasal medium (Gibco), 5% fetal calf serum (Cytiva, Mississauga, ON, Canada), 2% B27 supplement (Gibco), 1% penicillin/streptomycin (Gibco), and 1% 200 mM L-glutamine (Gibco)). Cells were plated at a density of 3.0 × 10^5^ cells/well onto 24-well CytoView MEA plates (Axion Biosystems, Atlanta, GA, USA) for electrophysiological recordings, 1.5 × 10^5^ cells/well onto Corning 24-well plates with 12 mm glass cover slips (Fisher Scientific, Ottawa, ON, Canada) for immunocytochemistry, and 1.0 × 10^6^ cells/well onto Corning 6-well plates for Western blotting. All plates were coated with 0.1 mg/mL poly-D-lysine (Sigma-Aldrich, Oakville, ON, Canada) and 2.5 µg/mL laminin (Sigma-Aldrich). Half-media changes were performed with serum-free media (Neurobasal Medium, 2% B27 supplement, 1% penicillin/streptomycin and 1% 200mM L-glutamine; Gibco) 3 hours after plating and every 3–4 days throughout the period of culture. Electrophysiology and immunocytochemistry experiments were carried out on day in vitro (DIV) 21, while cultures were maintained up to DIV 28 for Western blotting.

### 4.3. Drugs and Treatment of Cell Cultures

MAM was purchased from MRI Global (Kansas City, MO, USA). ILO (Sigma-Aldrich, Oakville, ON, Canada), HAL (Sigma-Aldrich, Oakville, ON, Canada), and CBD (Toronto Research Chemicals, North York, ON, Canada) were dissolved as stocks in DMSO to a concentration of 1 mM (ILO, HAL) or 10 mM (CBD). For all experiments, fresh serum free medium supplemented with compounds was added directly to well with cells. Final concentrations for ILO and HAL was 100 nM (0.02% DMSO) and 1 µM (0.02% DMSO) for CBD. These concentrations were based on previous studies [64,103]. 

### 4.4. MEA Recordings

All MEA readings were performed using the Maestro Edge multi-well MEA recorder (Axion Biosystems, Atlanta, GA, USA) and recorded with the Maestro system and Axion’s Integrated Studio (AxIS, Axion Biosystems, Atlanta, GA, USA) software configured to neuronal–spontaneous activity. Active electrodes were defined as >5 spikes/min across wells. Plates were maintained at 37 °C for the duration of recordings. Electrical activity was recorded for 10 min prior to drug treatments, as well as 5 min, 20 min, and 24 h post-drug administration. Recordings were taken at a sampling rate of 12.5 kHz, a gain of 1000 times, and a spike detection threshold of 6 times standard deviation. Burst detection criteria was defined as having a maximum inter-spike interval of 100 msec and a minimum of 5 spikes. Network burst detection criteria was set to a maximum inter-spike interval of 100 ms, a minimum of 50 spikes and at least 35% of active electrodes spiking simultaneously. Spike files were processed and analyzed using the NeuralMetric Tool software (Axion Biosystems, Atlanta, GA, USA). 

### 4.5. Immunocytochemistry

Immunocytochemistry was performed as done previously [104]. In each well, 500 µL of media was replaced with 8% paraformaldehyde and then incubated for 30 min at room temperature. After fixation, cells were washed three times in 1X phosphate-buffered saline (PBS) for 10 min and then blocked in 5% non-fat milk, 4% bovine serum albumin (BSA) (Thermo Fischer Scientific, Canada) and 0.1% Triton x100 (Fisher Scientific, Ottawa, ON, Canada) in 1X PBS for 60 min at room temperature. Cells were immunostained with a primary antibody for MAP2 (1:1000, Sigma-Aldrich, Oakville, ON, Canada), followed by secondary donkey anti-rabbit Alexa Fluor 594 (1:500, Invitrogen, Life Technologies, Burlington, ON, Canada), Alexa Fluor 488 phalloidin (100 nM, Invitrogen), and DAPI (1 µg/mL; Cell Signaling, Whitby, ON, Canada). Cover slips with cultured cortical networks were mounted on microscope slides with Prolong Gold (Invitrogen) overnight at 4 °C and subsequently imaged using a Nikon Eclipse Ti2 at 20 times magnification. 

### 4.6. Western Blotting

For Western blot analyses, cells were collected in ice-cold RIPA buffer (Sigma-Aldrich, Oakville, ON, Canada) with added phosphatase and protease inhibitors. Samples were centrifuged at 17,530× *g* for 15 min at 4 °C, supernatants were transferred to a fresh tube, and lysates were stored in aliquots at −80 °C until time of analysis. Protein concentrations were determined using the Bradford assay (Bradford, 1976). Protein samples (20 μg) were loaded onto a 10% sodium dodecyl sulfate (SDS) polyacrylamide gel for electrophoresis at 100 V using a Mini-PROTEAN Tetra cell system (Bio-Rad, Mississauga, ON, Canada). Proteins were then transferred at 25 V for 30 min onto polyvinylidene difluoride (PVDF) membrane (Bio-Rad, Mississauga, ON, Canada) using a Trans-Blot SD Turbo apparatus (Bio-Rad). Membranes were briefly washed in tris-buffered saline (TBS) with 0.1% Tween-20 (TBS-T) and then blocked in either 5% non-fat milk or 5% BSA (Fisher Scientific, Ottawa, ON, Canada) in TBS-T for 1 h at room temperature. Following blocking, membranes were washed twice with TBS-T and incubated overnight at 4 °C in primary antibody specific for either PSD-95 (1:1000, Cell Signaling, Whitby, ON, Canada), synapsin-1 (1:10,000,New England Biolabs, Whitby, ON, Canada), or β-actin (1:10,000, Abcam, Toronto, ON, Canada). Blots were washed three times with TBS-T, incubated with anti-rabbit horseradish peroxidase (HRP)-conjugated polyclonal secondary antibody (1:6000; Bio-Rad, Mississauga, ON, Canada) in milk or BSA for 1 h at room temperature, and then washed three times with TBS-T. Bands were visualized using enhanced chemiluminescence (ECL) (Bio-Rad) on a ChemiDoc MP imaging system (Bio-Rad, Mississauga, ON, Canada). 

### 4.7. Statistical Analysis

Prior to all analyses, a Shapiro–Wilk test was used to assess normality and differences in group variance assessed by Levene’s test. Baseline measurements for each experimental group were taken from all active electrodes across each plate. Following drug treatment, activity measures were calculated using data from all active electrodes in each of the 4 wells. The mean firing rate, number of bursts, inter-burst interval, and burst frequency were calculated using data from all active electrodes within a treatment group whereas number of network bursts and synchrony index were calculated using the mean from each well with more than 6 active electrodes. Baseline data was analyzed using a two-way analysis of variance (ANOVA) with sex and model as between-subject effects, and significant between group differences determined by Bonferroni or Games–Howell post-hoc tests as appropriate. Post-drug administration data were analyzed at each time point using a one-way ANOVA, with treatment as a between-subjects variable, followed by planned comparisons using Student’s *t*-test. Computations were performed using IBM SPSS 27 software and are expressed as mean ± SEM. Statistical significance was defined as *p* < 0.05.

## Data Availability

Data will be made available upon reasonable request.

## Abbreviations

CBD	Cannabidiol
HAL	Haloperidol
ILO	Iloperidone
MAM	Methylazoxymethanol Acetate
SAL	Saline
SZ	Schizophrenia
VEH	Vehicle
